# Spontaneous vs. Strategic Guilt: Guilt Communication in Repairing Trust with Different Severities of Violations

**DOI:** 10.3390/bs15081035

**Published:** 2025-07-30

**Authors:** Binghai Sun, Chuanyu Yang, Yuqi Feng, Shang Jin, Ningmeng Cao, Guoan Yue

**Affiliations:** 1 Zhejiang Normal University, Jinhua 321001, China; 2 School of Psychology, Zhejiang Normal University, Jinhua 321001, China; 3 College of Education, Zhejiang Normal University, Jinhua 321001, China

**Keywords:** spontaneous guilt communication, strategic guilt communication, severity of trust violation, trust repair

## Abstract

Previous research has predominantly focused on the impact of emotions on trust repair, yet has largely neglected how the manner of emotional communication influences this process. Centering on guilt—the emotion most commonly experienced by transgressors following a trust breach—this study examines whether spontaneous versus strategic guilt communication exerts differential effects on trust repair, and explores how the severity of the trust violation moderates this relationship. Study 1 compared the trust repair efficacy of spontaneous and strategic guilt communication. Study 2 tested the moderating role of violation severity. Results from Study 1 indicated that spontaneous guilt communication facilitated significantly greater trust repair than strategic communication. Study 2 further revealed that this advantage emerged only when the trust violation was severe. These findings advance theoretical understanding of guilt as a trust repair strategy and offer practical guidance for nurturing and restoring trust in interpersonal contexts, thereby promoting relational harmony.

## 1. Introduction

Trust violations are ubiquitous and impose substantial economic, emotional, and social costs on victims (Van der Werff et al., 2023; Yan & Wu, 2016); in extreme cases, they elicit destructive retaliation (Cao et al., 2024; Jahanzeb et al., 2020). Consequently, effective trust repair has become a central concern in organizational and interpersonal research (Sharma et al., 2023). Beyond verbal and behavioral reparative strategies (Lewicki & Brinsfield, 2017), scholars have increasingly emphasized the role of emotional communication, which Social Appraisal Theory (Van Kleef & Côté, 2022) conceptualizes as carriers of “social appraisal information.” Observers decode the transgressor’s emotions to infer responsibility attributions and behavioral legitimacy, thereby forming expectations about the transgressor’s future conduct.

Guilt, a prototypical post-transgression emotion, functions as a critical social mechanism linking trust violation to repair. Upon betraying trust, the trustee experiences acute guilt—an inherently moral emotion (Gangemi et al., 2025). Its primary social purpose is to restore trust by signaling remorse and a genuine intention to make amends (Nunney et al., 2022; Choi, 2022). In a trust game paradigm, Nunney et al. (2022) demonstrated that group communication of guilt significantly increased victims’ willingness to cooperate and forgive. Despite these insights, extant research has privileged the presence versus absence of guilt communication (e.g., the effectiveness of apologies) while largely overlooking how guilt is communicated—specifically, variations in expressive patterns.

Shore and Parkinson (2018) distinguish between spontaneous and strategic guilt communication based on intentionality. Spontaneous guilt communication arises when the trustee experiences genuine, unpremeditated guilt and transmits the emotion without conscious intent; observers infer the transgressor’s remorse through behavioral or paralinguistic cues rather than via deliberate disclosure. Strategic guilt communication, by contrast, entails an intentional effort to convey guilt to secure forgiveness, for instance, by overtly announcing remorse and promising restitution. Their findings demonstrate that spontaneous guilt yields superior trust repair outcomes relative to strategic communication. Complementarily, Hong et al. (2025) demonstrate that leaders who publicly communicate guilt elicit higher levels of follower forgiveness and organizational citizenship behavior. Collectively, these studies suggest that guilt communication—regardless of its underlying intentionality—exerts a consistent prosocial influence, facilitating both the restoration of damaged trust and the promotion of cooperative behavior.

Real-world trust violations span a continuum from minor deceptions to grave breaches of law and ethics (Yu, 2019; Sharma et al., 2023). Nunney et al. (2022) note that the efficacy of guilt communication is contingent on contextual factors such as group relational quality and prior behavioral history, and that its manifestations and effects vary across cultures. Furthermore, Polman et al. (2024) proposed a new perspective on emotions: the sensitivity of emotions to adaptation and updating in response to new environments. Consistent with this view, Henderson et al. (2020) observe that trust repair becomes progressively more arduous as violation severity increases. Raza et al. (2023) demonstrated that the severity of service failures exerts a moderating effect on the relationship between trust repair strategies and trust recovery. Within the context of institutional trust repair, minor service failures allow institutional trust repair strategies to yield more effective results in restoring trust. However, as service failure severity increases, this effect diminishes.

In summary, the current study aims to first examine the adaptability of the prosocial interpersonal effects of guilt communication within the context of Chinese culture. Study 1 aims to replicate and extend the findings of Shore and Parkinson (2018), who found that spontaneous guilt communication was more effective than strategic guilt communication in promoting trust repair. The multi-round trust game is better able to capture dynamic changes in trust relationships and provide a more accurate measure of trust repair than the single-round game. Therefore, Study 1 employed a multi-round trust game to investigate the effects of different guilt communication methods on trust repair, replicating Shore and Parkinson (2018). Second, the severity of trust violation can influence the effectiveness of guilt communication modes in trust repair. Building on the findings of Study 1, Study 2 aimed to explore whether the severity of trust violation moderates the effectiveness of different guilt communication modes in promoting trust repair.

## 2. Study 1

Study 1 investigates the causal impact of guilt communication mode on trust repair. Using a between-subjects design, participants were randomly assigned to one of three conditions: spontaneous guilt, strategic guilt, or control condition. Trust repair was operationalized as the amount invested by the trustor in a multi-round trust game. The present study hypothesizes that spontaneous guilt communication will elicit higher trust-repairing investments than strategic guilt communication.

### 2.1. Method

#### 2.1.1. Participants

The study employed a single-factor, between-subjects design with three levels. An a priori power analysis conducted in G*Power 3.1 indicated that 159 participants were required to detect a small effect (*f* = 0.25) with 80% power at α = 0.05. One hundred and fifty-nine non-psychology undergraduates (29 men, 18.2%; 130 women, 81.8%; *Mage* = 20.34, *SD* = 1.83) were recruited via online advertisements and randomly assigned to the spontaneous guilt (*n* = 53), strategic guilt (*n* = 53), or control (*n* = 53) conditions. All participants provided informed consent and received monetary compensation upon completion.

#### 2.1.2. Material

##### Trust Game

The study employed the trust game paradigm (Berg et al., 1995; Safra et al., 2022), which involves two types of players: the investor (trustor) and the investee (trustee). At the start of each round, the trustor is given an initial sum and can invest X units in the trustee. This investment is then tripled to 3X units for the trustee to receive, who decides whether to return any amount (from 0 to 3X units) to the trustor. Once the trustee makes this decision, the round ends. In this paradigm, the trustor’s investment amount reflects their level of trust in the trustee.

##### Propensity to Trust

The study employed the 4-item Trust Propensity Scale developed by Frazier et al. (2013) to assess participants’ generalized willingness to trust others. The items are: (1) “I usually trust people until they give me a reason not to trust them.” (2) “Trusting another person is not difficult for me.” (3) “My typical approach is to trust new acquaintances until they prove I should not trust them.” (4) “My tendency to trust others is high.” Responses were rated on a 5-point Likert scale (1 = strongly disagree, 5 = strongly agree), with higher summed scores indicating greater trust propensity. In the present sample, Cronbach’s α = 0.89, indicating excellent internal consistency.

##### Trait Forgiveness Scale

Trait forgiveness was measured using the 10-item Trait Forgiveness Scale (TFS; Berry & Worthington, 2001). The scale comprises five positively worded and five negatively worded items, rated on a 5-point Likert scale (1 = strongly disagree, 5 = strongly agree); higher total scores reflect a stronger disposition to forgive. Cronbach’s α = 0.75, indicating acceptable reliability.

#### 2.1.3. Procedure

Upon arrival, participants provided informed consent and basic demographic information before receiving standardized instructions for the trust game. The game assigns each participant the role of Trustor (A) for all rounds, while Trustee (B)’s responses are algorithmically determined. At the start of every round, A receives 10 monetary units (MUs) and may transfer any amount X (0 ≤ X ≤ 10) to B. The transferred sum is tripled to 3X (0 ≤ 3X ≤ 30), after which B—here simulated by the computer—decides what proportion Y (0% ≤ Y ≤ 100%) of 3X to return to A.

After confirming comprehension of the rules, participants completed eight consecutive trust game rounds with preprogrammed trustee returns designed to model trust formation, violation, and repair. Rounds 1–4 (trust-building): The trustee returned 50% of the tripled investment regardless of the amount sent. Rounds 5–6 (trust violation): No returns (0%) were provided. Rounds 7–8 (trust repair): The trustee returned 50% of the tripled investment.

Following the sixth round, we introduced the guilt communication manipulation. Participants viewed a message reporting the counterpart’s guilt over the preceding two rounds; the rating was fixed at 4 on a 5-point scale (1 = very slightly, 5 = extremely). In the spontaneous guilt condition, the message stated that this rating had been disclosed without the counterpart’s awareness. In the strategic guilt condition, participants were informed that the counterpart knowingly shared the rating. The control condition received no guilt information and proceeded directly to the next phase.

**Figure 1:**
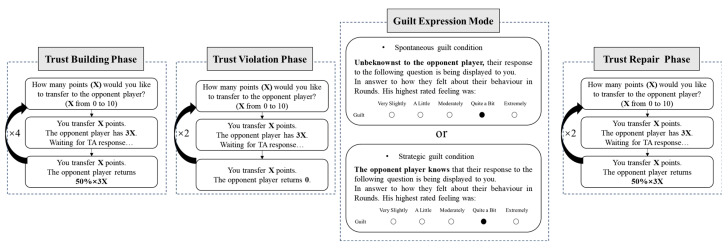
Procedure diagram of Study 1.

Immediately after the guilt communication manipulation, participants completed four manipulation check items adapted from Shore and Parkinson (2018). These assessed (1) perceived guilt (“To what extent does the other participant feel guilty?”), (2) perceived authenticity (“How genuine is the other participant’s emotional communication?”), (3) perceived intentionality (“Is the other participant’s guilt display a deliberate attempt to seek your forgiveness?”), and (4) perceived remorse (“How remorseful is the other participant?”). All items were rated on a 5-point scale (1 = not at all, 5 = very much). The complete procedure is depicted in Figure 1.

#### 2.1.4. Data Analysis

All analyses were conducted using SPSS 25. A single-factor between-subjects ANOVA examined how three guilt communication conditions (spontaneous, strategic, control) influenced trust repair efficacy, operationalized as the change in investment from Round 6 to Round 7 (ΔInvestment = Investment_7_ − Investment_6_). A one-way ANOVA tested the main effect of condition on ΔInvestment, and a repeated-measures ANOVA across Rounds 5–6 validated the effectiveness of the trust-violation manipulation. Normality was assessed via skewness, kurtosis, and histograms; all variables were normally distributed (see Supplementary Information).

### 2.2. Results

#### 2.2.1. Manipulation Check of Guilt Communication Mode

Manipulation checks for the guilt communication mode focused on four items: perceived guilt, perceived authenticity, perceived apology, and perceived intentionality, with their mean score serving as the indicator. The control condition was excluded from this analysis. Independent samples *t*-tests (see Table 1) revealed that the spontaneous guilt condition scored significantly higher on the mean score (3.06 ± 0.71) than the strategic guilt condition (2.68 ± 0.59), *t* = 2.98, *p* = 0.004, *Cohen’s d* = 0.58, confirming the successful manipulation of guilt communication mode.

#### 2.2.2. Trust Violation Verification

To verify the occurrence of trust violations, measurements were taken via investment amounts in the trust game and subjective reports. A repeated-measures ANOVA (See Table 2) on investment amounts (Rounds 5 and 6) revealed a significant main effect of rounds, *F* = 144.94, *p* < 0.001, *η_p_*^2^ = 0.482, with higher investments in Round 5 (7.62 ± 2.27) than in Round 6 (4.94 ± 2.51). Neither the main effect of guilt communication mode (*F* = 1.85, *p* = 0.161, *η_p_*^2^ = 0.023) nor the interaction of guilt communication mode and rounds (*F* = 0.52, *p* = 0.596, *η_p_*^2^ = 0.007) was significant. The study only found a significant main effect of the round and did not discover a significant interaction between the communication of guilt and the round. This result indicates that there is no difference in the severity of trust violation among the experimental groups.

#### 2.2.3. The Impact of Guilt Communication Modes on Trust Repair

A one-way ANOVA was performed on the investment repair value, controlling for gender, age, trust propensity, and forgiveness propensity. The results (Table 3) showed a significant main effect of guilt communication modes, *F* = 3.54, *p* = 0.032, *η_p_*^2^ = 0.044. Post-hoc comparisons indicated that the investment repair value was significantly higher in the spontaneous guilt condition (1.06 ± 2.58) than in the strategic guilt condition (0.12 ± 2.02, *p* = 0.045) and the control condition (−0.05 ± 2.35, *p* = 0.014). However, no significant difference was found between the strategic guilt condition and the control condition (*p* = 0.623). This suggests that spontaneous guilt communication is more effective for trust repair than strategic or no guilt communication.

### 2.3. Discussion

Study 1 employed a trust game paradigm to explore how spontaneous and strategic guilt communication from the trustee influences trust repair following a violation. The results indicated that spontaneous guilt communication yielded better trust repair than strategic guilt communication, aligning with previous findings (Shore & Parkinson, 2018). Prior studies have shown that the severity of trust violation impacts trust repair efficacy (Raza et al., 2023). Thus, Study 1 suggested that the severity of trust violation may also influence the effectiveness of guilt communication strategies in repairing trust. Building on these findings, Study 2 will examine how the severity of trust violation moderates the relationship between guilt communication types and trust repair.

## 3. Study 2

Study 2 employed a 2 (guilt communication mode: spontaneous vs. strategic) × 3 (trust violation severity: high vs. moderate vs. low) between-subjects factorial design to test whether violation severity moderates the efficacy of guilt communication in restoring trust. Study 2 hypothesized that (a) under low and moderate severity, both spontaneous and strategic guilt would facilitate repair to a similar extent, whereas (b) under high severity, spontaneous communication would yield significantly greater trust repair than strategic communication.

### 3.1. Method

#### 3.1.1. Participants

The study employed a 2 (guilt communication mode: spontaneous vs. strategic) × 3 (trust violation severity: high vs. moderate vs. low) between-subjects design. An a priori power analysis (G*Power 3.1) indicated that 158 participants were required to detect a small effect (*f* = 0.25) with 80% power at α = 0.05. In total, 160 non-psychology undergraduates (39 men, 24.4%; 121 women, 75.6%; *Mage* = 20.36, *SD* = 1.77) were recruited online and randomly assigned to conditions. All provided informed consent and received monetary compensation upon completion.

#### 3.1.2. Material

Study 2 replicated the eight-round structure of Study 1 (Rounds 1–4: trust-building; Rounds 5–6: trust violation; Rounds 7–8: trust repair) and retained all dependent measures. Severity of violation was manipulated solely in Rounds 5–6 by varying trustee returns: 0% of 3X in the high-severity condition, 20% of 3X in the moderate-severity condition, and 40% of 3X in the low-severity condition (Qin, 2015).

#### 3.1.3. Data Analysis

Analyses were performed with SPSS 25. Employing a 2 (guilt communication mode) × 3 (trust violation severity) between-subjects design, we first verified the guilt communication manipulation with an independent-samples *t*-test. A repeated-measures ANOVA across Rounds 5–6 confirmed the effectiveness of the violation manipulation. Finally, a 2 × 3 factorial ANOVA was employed to investigate the impact of guilt communication mode and the severity of trust violation on individual trust repair. Prior to conducting the formal analysis, the study first performed a normality test. The study employed skewness, kurtosis, and histograms of the data distribution to conduct the normality test. The results indicated that the data conformed to a normal distribution (details can be found in the Supplementary Information).

### 3.2. Results

#### 3.2.1. Manipulation Check of Guilt Communication Mode

An independent-samples *t*-test (Table 4) revealed that the mean score for the manipulation check items was significantly higher in the spontaneous guilt condition (3.20 ± 0.60) than in the strategic guilt condition (2.74 ± 0.69), *t* = 4.47, *p* < 0.001, *Cohen’s d* = 0.71. This indicates that the manipulation of guilt communication mode was effective.

#### 3.2.2. Trust Violation Verification

A repeated-measures ANOVA (Table 5) on investment amounts for rounds 5 and 6 revealed a significant main effect of rounds, *F* = 136.32, *p* < 0.001, *η_p_*^2^ = 0.470, with higher investments in round 5 (7.85 ± 2.25) than in round 6 (5.79 ± 2.43). No significant main effect of guilt communication modes (*F* = 0.17, *p* = 0.682, *η_p_*^2^ = 0.001) or the interaction of guilt communication modes and rounds (*F* = 0.30, *p* = 0.585, *η_p_*^2^ = 0.002) was found. Additionally, a significant main effect of violation severity was observed (*F* = 6.86, *p* = 0.001, *η_p_*^2^ = 0.082). Post-hoc analyses indicated that the severity of trust violation was significantly lower under low-severity conditions compared to moderate- and high-severity conditions. However, there is no difference in the severity of trust violation between the medium- and high-severity conditions.

#### 3.2.3. The Impact of Guilt Communication and Trust Violation Degree on Trust Repair

A 2 × 3 ANOVA (Table 6) was performed with investment repair value as the dependent variable, guilt communication mode and violation severity as independent variables, and gender, age, and trust propensity as covariates. The results showed a significant main effect of guilt communication mode, *F* = 6.35, *p* = 0.013, *η_p_*^2^ = 0.041, with higher investment repair in spontaneous guilt condition (0.95 ± 2.48) than strategic guilt condition (0.01 ± 2.21, *p* = 0.013). The interaction between guilt communication mode and violation degree was marginally significant for investment repair, *F* = 2.90, *p* = 0.058, *η_p_*^2^ = 0.037. Simple-effects analysis showed that under high violation degree, spontaneous guilt condition led to greater investment repair (1.78 ± 2.46) than strategic guilt condition (−0.52 ± 2.79, *F* = 11.58, *p* = 0.001, *η_p_*^2^ = 0.072). No significant differences were found between the two guilt communication modes under low or moderate violation degree. The main effect of violation degree was non-significant, *F* = 1.84, *p* = 0.163, *η_p_*^2^ = 0.024.

### 3.3. Discussion

Study 2 employed a 2 (guilt communication mode: spontaneous vs. strategic) × 3 (trust violation severity: high vs. moderate vs. low) between-subjects factorial design to test whether violation severity moderates the efficacy of guilt communication in restoring trust. Study 2 hypothesized that (a) under low and moderate severity, both spontaneous and strategic guilt communication would facilitate trust repair to a similar extent, whereas (b) under high severity, spontaneous guilt communication would yield significantly greater trust repair than strategic communication.

## 4. General Discussion

Replicating Shore and Parkinson (2018), Study 1 demonstrated that spontaneous guilt outperforms strategic guilt in restoring trust. Study 2 revealed that this advantage emerges only when the violation is severe. Under high-severity conditions, spontaneous guilt elicited significantly larger post-breach investments than strategic guilt, whereas the two modes yielded equivalent outcomes under low or moderate severity. These findings underscore that the efficacy of guilt-based repair strategies is contingent on the severity of the transgression.

### 4.1. The Trust Repair Advantage of Spontaneous Guilt Communication

Social Appraisal Theory and the Emotions as Social Information (EASI) model (Van Kleef & Côté, 2022; van Kleef, 2009) posit that the prosocial interpersonal effects of guilt communication operate through affective and inferential processes. Regarding affective processes, guilt communication can elicit sympathy and empathy in observers, allowing them to perceive the expresser’s remorse and sincerity (Van Kleef et al., 2012). Through inferential processes, observers derive the expresser’s negative evaluation of the transgression and intention to make amends—including attributions of responsibility and expectations of future behavior—thereby enhancing trust in the expresser (van Doorn et al., 2015). In short, guilt communication transmits signals of sincerity and intent to change, reshaping the recipient’s perception of the expresser’s trustworthiness and thereby determining subsequent cooperative or competitive behaviors.

Study 1 confirmed that spontaneous guilt communication more effectively promotes trust repair than strategic guilt communication, aligning with the findings of Shore and Parkinson (2018). Grounded in Social Appraisal Theory and the Emotions as Social Information (EASI) model, the affective process suggests that observers perceive spontaneous guilt as more sincere due to its lack of intentionality—consistent with research showing that “an honest apology is more likely to promote forgiveness” (Forster et al., 2021; Mu & Bobocel, 2019). Conversely, strategic guilt is readily interpreted as manipulative, reducing individuals’ willingness to rebuild trust. Via the inferential process, strategic guilt may lead the trustor to attribute the violation to intentional wrongdoing, intensifying feelings of betrayal (Lewicki & Brinsfield, 2017). Spontaneous guilt, by contrast, is more likely to be attributed to unintentional action, thereby reducing barriers to repair (Klackl et al., 2013).

### 4.2. The Severity of Trust Violation Moderates the Trust Repair Advantage Conferred by Spontaneous Guilt Communication

Study 2 revealed that the severity of a trust breach moderates the efficacy of guilt communication strategies. Under high-severity violations, spontaneous guilt communication is more effective than strategic guilt communication. Under low- or moderate-severity violations, no significant difference emerged between the two. This indirectly corroborates the theory that the efficacy of trust repair strategies is contingent upon the violation context. It should be noted that the moderating role of trust violation severity remains to be further validated. The current findings indicate that this moderation effect only approached statistical significance (*p* = 0.058). However, subsequent simple effect analyses revealed differences in trust restoration between spontaneous and strategic guilt communication across different violation severity levels.

In high-severity violations, the victim experiences stronger negative emotions and exhibits lower trust in any repair attempt. Under such circumstances, victims rely more heavily on “sincerity cues” to rebuild trust. At this point, spontaneous guilt communication, perceived as more authentic, is more readily accepted and thus effectively restores trust. Under low- or moderate-severity violations, victims experience milder negative emotions and retain higher baseline trust, rendering them less sensitive to the mode of guilt communication. Consequently, victims may be more receptive to diverse repair strategies, encompassing both spontaneous and strategic guilt communication. Therefore, the repair outcomes of the two modes did not differ significantly.

### 4.3. Theoretical and Practical Implications

Using a multi-round trust game paradigm, the study investigated the impact of different guilt communication modes (spontaneous vs. strategic guilt) on trust repair and the moderating role of the severity of trust violation. The study found that spontaneous guilt communication is more effective in repairing trust than strategic guilt communication. The finding offers a new perspective on the theory of trust repair and holds significant practical implications.

#### 4.3.1. Theoretical Implications

The study makes two theoretical contributions. First, by focusing on emotional communication modes rather than repair strategies per se, this study addresses a neglected dimension of trust repair. Distinguishing spontaneous from strategic guilt reveals that communication patterns differentially influence trust restoration, thereby extending the trust repair framework (Lewicki & Brinsfield, 2017). Second, this study demonstrates that violation severity moderates the guilt repair relationship, underscoring trust restoration as a dynamic process whose efficacy is contingent on situational extremity. These findings offer novel evidence for the role of emotional communication in trust repair (Shore & Parkinson, 2018) and delineate boundary conditions for the Emotions as Social Information model.

#### 4.3.2. Practical Implications

This study provides specific guidance on “non-strategic sincere communication” for trust repair, emphasizing the natural expression of authentic emotions rather than deliberate imitation of spontaneity. Additionally, the study highlights the adoption of differentiated repair strategies according to the severity of the violation to enhance practical applicability. Specifically, in high-severity violation scenarios (e.g., major betrayals), the repairing party should convey remorse by actively assuming consequences and continuously compensating for losses through tangible actions, rather than relying on pre-scripted statements or performative apologies. In low-severity violation scenarios (e.g., minor errors), even a brief apology (e.g., directly acknowledging the oversight) can achieve repair, without excessive deliberate design. This tiered strategy not only avoids the issue of “blindly pursuing sincerity at the expense of efficiency” but also mitigates the risk of “over-strategizing and thereby losing trust.” It thus offers a practical trust repair framework for both individuals (e.g., in conflicts within intimate relationships) and organizations (e.g., in corporate crisis management).

### 4.4. Limitations and Future Directions

Using a multi-round trust game paradigm, this study examines the impact of guilt communication modes on trust repair and the moderating effect of trust violation degree. While providing new perspectives for trust repair theory and practice, it has limitations that need improvement in future research.

First, the singularity of emotional types. this study focused solely on guilt, without comparing how expression patterns of other negative emotions (e.g., regret, remorse) affect trust repair. Different emotions have distinct core motivational underpinnings. For example, Guilt is a negative emotion arising from regret over improper behavior and a desire to correct it, while shame is a negative emotion stemming from behavior failing to meet social expectations, and their expression patterns may yield different effects (Ghorbani et al., 2013). Future research could incorporate diverse emotional types to explore whether “emotional characteristics (e.g., relationship-oriented vs. behavior-oriented) interact with expression patterns to influence trust repair,” thereby enriching the theoretical framework of emotional repair strategies.

Second, limitations in sample representativeness and gender ratio imbalance. This study’s samples were exclusively college students, with a significantly skewed female ratio (81.76% in Study 1 and 75.62% in Study 2), potentially restricting findings’ generalizability. On one hand, college students’ trust decisions, shaped by limited social experience, differ from those of professionals or community residents. On the other hand, the skewed ratio may obscure gender differences in trust repair—though gender showed no significant effect as a covariate here, existing research notes potential differences (e.g., women prioritize sincerity in emotional expression, men focus more on behavioral compensation). Such differences may limit findings’ applicability across genders. Future research should expand sample sources (e.g., diverse ages and occupations), balance gender ratios, and examine the moderating role of demographic variables (e.g., gender) via subgroup analysis.

Third, the study uses a standardized trust game paradigm, which enables precise variable control but differs from real-world trust dynamics. Real-world trust repair involves more complex emotional bonds, longer timeframes, and more external influences (e.g., public opinion, third-party intervention). For example, “instrumental trust”—measured via “investment amount” in the laboratory—may respond differently to guilt expression patterns than “emotional trust” rooted in real-world emotional bonds. Future research should design contexts with higher ecological validity (e.g., simulating workplace transgressions, longitudinally tracking intimate relationship betrayals) and distinguish repair differences between instrumental and emotional trust to enhance the findings’ practical applicability.

Finally, regarding constraints on generality: Using Simons et al.’s (2017) framework, this study’s findings are bounded by the following. At the population level, they primarily generalize to young college students; applicability to other age groups (e.g., middle-aged individuals) or cultural contexts (e.g., collectivist vs. individualist cultures) is untested. Cross-cultural variations may exist in interpreting “spontaneity” and “strategicity” in emotional expression—for example, in collectivist cultures, deliberate guilt expressions might be seen as “valuing relationships” rather than “manipulation.” At the situational level, findings are limited to “trust repair in one-time or short-term interactions” and may not extend to long-term cooperative relationships (e.g., marital or partnership bonds), which involve more complex historical interactions and relational commitments. Methodologically, using “investment amount” in the trust game may not fully capture the multidimensionality of real-world trust repair (e.g., differing repair rates of cognitive and emotional trust).

## 5. Conclusions

Guilt communication mode significantly influences trust repair: Spontaneous guilt consistently outperforms strategic guilt, but the advantage is moderated by violation severity. When violations are severe, spontaneous guilt yields substantially greater repair; under moderate or low severity, the two modes produce equivalent outcomes.

## Figures and Tables

**Table 1:** Manipulation Test Results of Guilt Communication Mode in Study 1.

Group	*M ± SD*	*t*	*p*	*d*
Spontaneous Guilt Condition	3.06 ± 0.71	2.98 **	0.004	0.58
Strategic Guilt Condition	2.68 ± 0.59			

Note: ** *p* < 0.01.

**Table 2:** Manipulation Test Results of Trust Violation in Study 1.

	*SS*	*df*	*MS*	*F*	*p*	*η_p_* ^2^
Guilt Communication Modes	27.55	2	13.78	1.85	0.161	0.023
Rounds	570.68	1	570.69	144.94 ***	<0.001	0.482
Guilt Communication Modes × Rounds	4.09	2	2.05	0.52	0.596	0.007

Note: *** *p* < 0.001.

**Table 3:** ANOVA Results for Differences in Investment Repair Values (N = 159).

	*SS*	*df*	*MS*	*F*	*p*	*η_p_* ^2^
Guilt Communication Modes	37.39	2	18.70	3.54 *	0.032	0.044
Covariate						
Gender	25.10	1	25.10	4.75 *	0.031	0.030
Age	0.87	1	0.87	0.16	0.686	0.001
Trust Propensity	9.73	1	9.37	1.84	0.177	0.012
Forgiveness Propensity	3.91	1	3.91	0.74	0.391	0.005

Note: * *p* < 0.05.

**Table 4:** Manipulation Test Results of Guilt Communication Mode in Study 2.

Group	*M ± SD*	*t*	*p*	*d*
Spontaneous Guilt Condition	3.20 ± 0.60	4.47 ***	<0.001	0.71
Strategic Guilt Condition	2.74 ± 0.69			

Note: *** *p* < 0.001.

**Table 5:** Manipulation Test Results of Trust Violation in Study 2.

	*SS*	*df*	*MS*	*F*	*p*	*η_p_* ^2^
Guilt Communication Modes	1.25	1	1.25	0.17	0.682	0.001
Violation Severity	101.60	2	50.80	6.86	0.001	0.082
Rounds	332.86	2	332.32	136.32	<0.001	0.047
Guilt Communication Modes × Rounds	0.73	1	0.73	0.30	0.585	0.002
Guilt Communication Modes × Violation Severity	24.02	2	12.01	1.62	0.201	0.021
Violation Severity × Rounds	91.08	2	445.54	18.65	<0.001	0.195
Guilt Communication Modes × Violation Severity × Rounds	2.44	2	1.22	0.50	0.608	0.006

**Table 6:** The Impact of Guilt Communication and Trust Violation Degree on Trust Repair (N = 106).

	*SS*	*df*	*MS*	*F*	*p*	*η_p_* ^2^
Guilt Communication Modes	33.44	1	33.44	6.35 *	0.013	0.041
Trust Violation Severity	19.37	2	9.69	1.84	0.163	0.024
Guilt Communication Modes × Trust Violation Severity	30.57	2	15.29	2.90	0.058	0.037
Covariate						
Gender	5.29	1	5.29	1.00	0.318	0.007
Age	4.24	1	4.24	0.81	0.371	0.005
Trust Propensity	16.79	1	16.79	3.19	0.076	0.021
Forgiveness Propensity	0.73	1	0.73	0.14	0.711	0.001

Note: * *p* < 0.05.

## Data Availability

The data that support the findings of this study are available from the corresponding author upon reasonable request.

## Supplementary Materials

The following supporting information can be downloaded at: https://www.mdpi.com/article/10.3390/bs15081035/s1.
