# Supplementary material for: Spontaneous vs. Strategic Guilt: Guilt Communication in Repairing Trust with Different Severities of Violations
Source: Behav Sci (Basel). 2025 Jul 30;15(8):1035. doi: 10.3390/bs15081035 (PMC12383218; doi:10.3390/bs15081035)
Supplement: Supplementary file 1 [file behavsci-15-01035-s001.zip › behavsci-3652530-supplementary.pdf]

Supplementary Information

Part 1: The Assumption of Normality in Study 1 and Study 2

SI Table S1 Skewness and Kurtosis of Data in Study 1 and Study 2

	Study 1	Study 2
Skewness	-.014(.192)	-.253(.192)
Z-score of skewness	-.073	-1.318
Kurtosis	.509(.383)	1.920(.381)
Z-score of Kurtosis	1.329	5.039

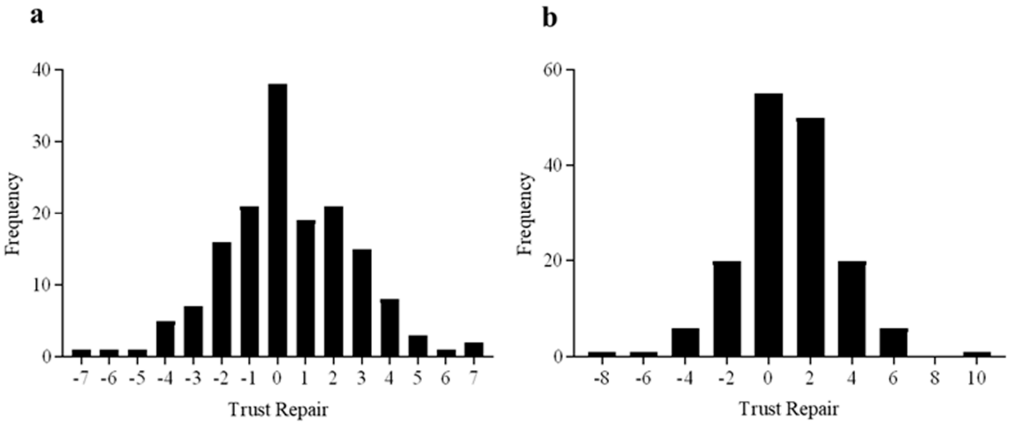

SI Figure S1 Histogram of Data Distribution. Figure 1a was the histogram of data distribution for Study 1; Figure 1b was the histogram of data distribution for Study 2.
